# *trans*-Dichloridobis(quinoline-κ*N*)platinum(II)

**DOI:** 10.1107/S1600536812013608

**Published:** 2012-04-04

**Authors:** Kwang Ha

**Affiliations:** a School of Applied Chemical Engineering, The Research Institute of Catalysis, Chonnam National University, Gwangju 500-757, Republic of Korea

## Abstract

In the title complex, *trans*-[PtCl_2_(C_9_H_7_N)_2_], the Pt^II^ ion is four-coordinated in an essentially square-planar coordination environment defined by two N atoms from two quinoline (qu) ligands and two Cl^−^ anions. The Pt atom is located on an inversion centre and thus the asymmetric unit contains one half of the complex; the PtN_2_Cl_2_ unit is exactly planar. The dihedral angle between the PtN_2_Cl_2_ unit and the quinoline ligand is 85.1 (1)°. In the crystal, the complex mol­ecules are stacked into columns along the *b* axis. In the columns, several inter­molecular π–π inter­actions between the six-membered rings are present, the shortest ring centroid–centroid distance being 3.733 (5) Å between pyridine rings.

## Related literature

For the crystal structure of (H-qu)_2_[PtCl_6_]·2H_2_O, see: Ha (2012*a* ▶). For the crystal structures of the related Pt^II^ complexes *cis*-[PtCl_2_(qu)_2_]_._0.25DMF (DMF = *N,N*-dimethyl­formamide) and *cis*-[PtCl_2_(qu)_2_]^.^CH_3_NO_2_, see: Davies *et al.* (2001 ▶); Ha (2012*b* ▶).
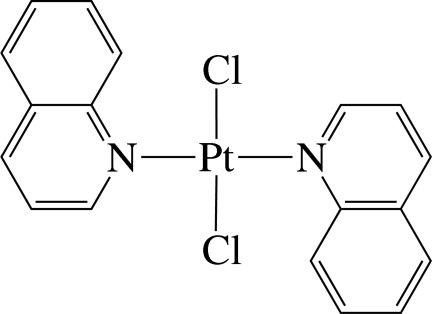


## Experimental

### 

#### Crystal data

[PtCl_2_(C_9_H_7_N)_2_]
*M* *_r_* = 524.30
Monoclinic, 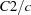

*a* = 16.3722 (18) Å
*b* = 6.9543 (7) Å
*c* = 16.0422 (17) Å
β = 118.684 (2)°
*V* = 1602.4 (3) Å^3^
*Z* = 4
Mo *K*α radiation
μ = 9.09 mm^−1^
*T* = 200 K
0.21 × 0.08 × 0.07 mm

#### Data collection

Bruker SMART 1000 CCD diffractometer
Absorption correction: multi-scan (*SADABS*; Bruker, 2000 ▶) *T*_min_ = 0.596, *T*_max_ = 1.000
4630 measured reflections
1569 independent reflections
1025 reflections with *I* > 2σ(*I*)
*R*_int_ = 0.053

#### Refinement

*R*[*F* ^2^ > 2σ(*F* ^2^)] = 0.034
*wR*(*F* ^2^) = 0.080
*S* = 0.97
1569 reflections
106 parameters
H-atom parameters constrained
Δρ_max_ = 1.74 e Å^−3^
Δρ_min_ = −0.97 e Å^−3^

### 

Data collection: *SMART* (Bruker, 2000 ▶); cell refinement: *SAINT* (Bruker, 2000 ▶); data reduction: *SAINT*; program(s) used to solve structure: *SHELXS97* (Sheldrick, 2008 ▶); program(s) used to refine structure: *SHELXL97* (Sheldrick, 2008 ▶); molecular graphics: *ORTEP-3* (Farrugia, 1997 ▶) and *PLATON* (Spek, 2009 ▶); software used to prepare material for publication: *SHELXL97*.

## Supplementary Material

Crystal structure: contains datablock(s) global, I. DOI: 10.1107/S1600536812013608/ng5260sup1.cif

Structure factors: contains datablock(s) I. DOI: 10.1107/S1600536812013608/ng5260Isup2.hkl

Additional supplementary materials:  crystallographic information; 3D view; checkCIF report

## Figures and Tables

**Table:** 

Pt1—N1	2.036 (6)
Pt1—Cl1	2.297 (2)

**Table:** 

N1—Pt1—Cl1	89.40 (18)

**Fig. 1.**
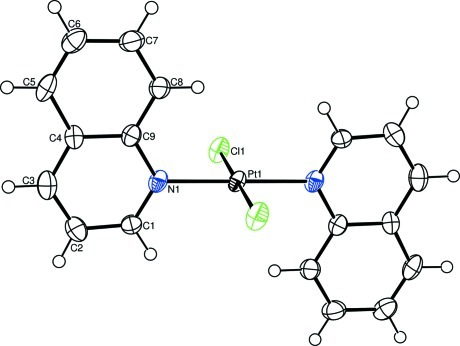
A view of the molecular structure of the title complex, with displacement ellipsoids drawn at the 50% probability level and the atom numbering. Unlabelled atoms are related to the reference atoms by the (-x, 1 - y, -z) symmetry transformation.

**Fig. 2.**
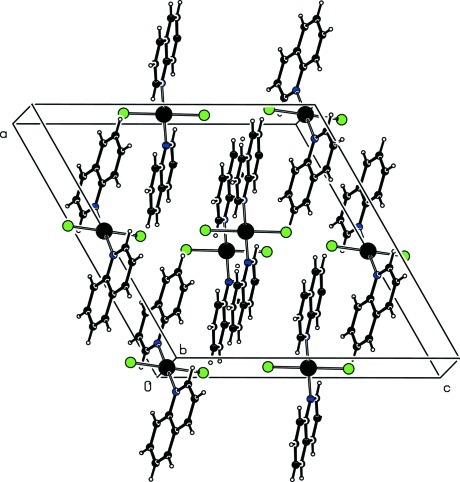
A view of the unit-cell contents of the title complex.

## supplementary crystallographic information

### Comment

The title complex, [PtCl_2_(qu)_2_] (qu = quinoline), was unexpected obtained as a byproduct from the reaction of K_2_PtCl_6_ with qu. The main product of the rection was found as the Pt^IV^ complex, (H-qu)_2_[PtCl_6_]^.^2H_2_O, and its crystal structure has been previously reported (Ha, 2012*a*). It seems that the Pt^IV^ ion reduced partially to the Pt^II^ ion in the reaction.

In the complex, the Pt^II^ ion is four-coordinated in an essentially square-planar coordination environment defined by two N atoms from two qu ligands and two Cl^-^ anions (Fig. 1 and Table 1). The Cl atoms are in *trans* conformation with respect to each other. By contrast, in the analogous Pt^II^ complexes [PtCl_2_(qu)_2_]^.^0.25DMF (DMF = *N,N*-dimethylformamide) (Davies *et al.*, 2001) and [PtCl_2_(qu)_2_]^.^CH_3_NO_2_ (Ha, 2012*b*), the Cl atoms are in *cis* conformation. The *cis*-Pt^II^ complexes were synthesized from the reaction of K_2_PtCl_4_ with qu.

The Pt atom is located on an inversion centre, and thus the asymmetric unit contains one half of the complex; the PtN_2_Cl_2_ unit is exactly planar. The nearly planar qu ligands, with a maximum deviation of 0.012 (7) Å from the least-squares plane, are parallel. The dihedral angle between the PtN_2_Cl_2_ unit and qu ligand is 85.1 (1)°. The Cl atoms are almost perpendicular to the qu planes, with the bond angle of <N1—Pt1—Cl1 = 89.40 (18)°. In the crystal, the complex molecules are arranged in a V-shaped packing pattern and stacked into two distinct columns along the *b* axis (Fig. 2). In the columns, several intermolecular π-π interactions between the six-membered rings are present, the shortest ring centroid-centroid distance being 3.733 (5) Å between pyridine rings.

### Experimental

The single crystals of the title complex were obtained as a byproduct from the reaction of K_2_PtCl_6_ (0.2432 g, 0.500 mmol) with quinoline (0.1569 g, 1.215 mmol) in H_2_O (30 ml). After refluxing of the reaction mixture for 3 h, the formed brown precipitate was removed by filtration, and the solvent of the filtrate was evaporated. The residue was washed with H_2_O/acetone (1:5) and dried at 50 °C, to give a yellow powder (0.2072 g) (Ha, 2012*a*). Crystals suitable for X-ray analysis were obtained by slow evaporation at 60 °C from an *N,N*-dimethylformamide (DMF) solution, which was obtained after filtration of the product over the solid-phase extraction column (4 ml) with silica (200 mg).

### Refinement

H atoms were positioned geometrically and allowed to ride on their respective parent atoms: C—H = 0.95 Å with *U*_iso_(H) = 1.2*U*_eq_(C). The highest peak (1.74 e Å^-3^) and the deepest hole (-0.97 e Å^-3^) in the difference Fourier map are located 1.10 Å and 1.51 Å, respectively, from the atoms Pt1 and N1.

### Crystal data

**Table:**

[PtCl_2_(C_9_H_7_N)_2_]	*F*(000) = 992
*M**_r_* = 524.30	*D*_x_ = 2.173 Mg m^−^^3^
Monoclinic, *C*2/*c*	Mo *K*α radiation, λ = 0.71073 Å
Hall symbol: -C 2yc	Cell parameters from 2043 reflections
*a* = 16.3722 (18) Å	θ = 2.8–25.9°
*b* = 6.9543 (7) Å	µ = 9.09 mm^−^^1^
*c* = 16.0422 (17) Å	*T* = 200 K
β = 118.684 (2)°	Block, yellow
*V* = 1602.4 (3) Å^3^	0.21 × 0.08 × 0.07 mm
*Z* = 4	

### Data collection

**Table:**

Bruker SMART 1000 CCD diffractometer	1569 independent reflections
Radiation source: fine-focus sealed tube	1025 reflections with *I* > 2σ(*I*)
Graphite monochromator	*R*_int_ = 0.053
φ and ω scans	θ_max_ = 26.0°, θ_min_ = 2.9°
Absorption correction: multi-scan (*SADABS*; Bruker, 2000)	*h* = −20→17
*T*_min_ = 0.596, *T*_max_ = 1.000	*k* = −8→8
4630 measured reflections	*l* = −19→19

### Refinement

**Table:**

Refinement on *F*^2^	Primary atom site location: structure-invariant direct methods
Least-squares matrix: full	Secondary atom site location: difference Fourier map
*R*[*F*^2^ > 2σ(*F*^2^)] = 0.034	Hydrogen site location: inferred from neighbouring sites
*wR*(*F*^2^) = 0.080	H-atom parameters constrained
*S* = 0.97	*w* = 1/[σ^2^(*F*_o_^2^) + (0.0357*P*)^2^] where *P* = (*F*_o_^2^ + 2*F*_c_^2^)/3
1569 reflections	(Δ/σ)_max_ < 0.001
106 parameters	Δρ_max_ = 1.74 e Å^−^^3^
0 restraints	Δρ_min_ = −0.97 e Å^−^^3^

### Special details

**Table:**

Geometry. All e.s.d.'s (except the e.s.d. in the dihedral angle between two l.s. planes) are estimated using the full covariance matrix. The cell e.s.d.'s are taken into account individually in the estimation of e.s.d.'s in distances, angles and torsion angles; correlations between e.s.d.'s in cell parameters are only used when they are defined by crystal symmetry. An approximate (isotropic) treatment of cell e.s.d.'s is used for estimating e.s.d.'s involving l.s. planes.
Refinement. Refinement of *F*^2^ against ALL reflections. The weighted *R*-factor *wR* and goodness of fit *S* are based on *F*^2^, conventional *R*-factors *R* are based on *F*, with *F* set to zero for negative *F*^2^. The threshold expression of *F*^2^ > σ(*F*^2^) is used only for calculating *R*-factors(gt) *etc*. and is not relevant to the choice of reflections for refinement. *R*-factors based on *F*^2^ are statistically about twice as large as those based on *F*, and *R*- factors based on ALL data will be even larger.

### Fractional atomic coordinates and isotropic or equivalent isotropic displacement parameters (Å^2^)

**Table:**

	*x*	*y*	*z*	*U*_iso_*/*U*_eq_	
Pt1	0.0000	0.5000	0.0000	0.02655 (16)	
Cl1	−0.01185 (14)	0.3931 (3)	0.12923 (14)	0.0373 (5)	
N1	0.1091 (4)	0.3178 (9)	0.0354 (4)	0.0270 (15)	
C1	0.0927 (5)	0.1473 (11)	−0.0038 (5)	0.0297 (19)	
H1	0.0304	0.1167	−0.0492	0.036*	
C2	0.1608 (6)	0.0079 (12)	0.0170 (6)	0.0353 (18)	
H2	0.1455	−0.1133	−0.0141	0.042*	
C3	0.2506 (6)	0.0500 (11)	0.0836 (6)	0.036 (2)	
H3	0.2986	−0.0426	0.0998	0.043*	
C4	0.2710 (5)	0.2304 (11)	0.1273 (5)	0.0247 (17)	
C5	0.3626 (5)	0.2867 (13)	0.1961 (5)	0.036 (2)	
H5	0.4125	0.1982	0.2142	0.043*	
C6	0.3801 (6)	0.4626 (12)	0.2362 (6)	0.036 (2)	
H6	0.4416	0.4970	0.2821	0.043*	
C7	0.3073 (6)	0.5943 (14)	0.2100 (6)	0.037 (2)	
H7	0.3201	0.7184	0.2382	0.044*	
C8	0.2177 (6)	0.5482 (11)	0.1443 (6)	0.031 (2)	
H8	0.1691	0.6393	0.1275	0.037*	
C9	0.1986 (5)	0.3668 (11)	0.1025 (5)	0.0257 (18)	

### Atomic displacement parameters (Å^2^)

**Table:**

	*U*^11^	*U*^22^	*U*^33^	*U*^12^	*U*^13^	*U*^23^
Pt1	0.0162 (2)	0.0315 (2)	0.0279 (2)	0.0031 (3)	0.00741 (16)	0.0021 (3)
Cl1	0.0322 (11)	0.0474 (13)	0.0335 (10)	0.0094 (11)	0.0169 (9)	0.0103 (11)
N1	0.016 (3)	0.031 (4)	0.033 (3)	0.000 (3)	0.011 (3)	0.003 (3)
C1	0.023 (4)	0.027 (5)	0.033 (4)	−0.003 (4)	0.009 (4)	−0.005 (4)
C2	0.041 (5)	0.025 (4)	0.045 (4)	0.001 (5)	0.025 (4)	0.001 (5)
C3	0.034 (5)	0.037 (6)	0.045 (5)	0.011 (4)	0.025 (4)	0.011 (4)
C4	0.025 (4)	0.023 (4)	0.033 (4)	0.002 (3)	0.018 (4)	0.007 (4)
C5	0.019 (4)	0.050 (6)	0.036 (5)	0.008 (4)	0.011 (4)	0.009 (4)
C6	0.025 (4)	0.054 (7)	0.029 (4)	0.000 (4)	0.013 (4)	0.000 (4)
C7	0.030 (5)	0.042 (5)	0.033 (4)	−0.005 (4)	0.011 (4)	−0.008 (4)
C8	0.023 (4)	0.036 (6)	0.034 (4)	0.001 (3)	0.014 (4)	−0.005 (4)
C9	0.022 (4)	0.030 (5)	0.026 (4)	0.002 (4)	0.013 (4)	0.004 (4)

### Geometric parameters (Å, º)

**Table:**

Pt1—N1^i^	2.036 (6)	C3—H3	0.9500
Pt1—N1	2.036 (6)	C4—C9	1.418 (10)
Pt1—Cl1^i^	2.297 (2)	C4—C5	1.425 (10)
Pt1—Cl1	2.297 (2)	C5—C6	1.347 (10)
N1—C1	1.308 (9)	C5—H5	0.9500
N1—C9	1.382 (9)	C6—C7	1.398 (12)
C1—C2	1.392 (10)	C6—H6	0.9500
C1—H1	0.9500	C7—C8	1.371 (11)
C2—C3	1.371 (12)	C7—H7	0.9500
C2—H2	0.9500	C8—C9	1.392 (11)
C3—C4	1.398 (10)	C8—H8	0.9500
			
N1^i^—Pt1—N1	180.0	C3—C4—C9	119.6 (7)
N1^i^—Pt1—Cl1^i^	89.40 (18)	C3—C4—C5	123.0 (8)
N1—Pt1—Cl1^i^	90.60 (18)	C9—C4—C5	117.4 (7)
N1^i^—Pt1—Cl1	90.60 (18)	C6—C5—C4	121.5 (8)
N1—Pt1—Cl1	89.40 (18)	C6—C5—H5	119.2
Cl1^i^—Pt1—Cl1	180.00 (10)	C4—C5—H5	119.2
C1—N1—C9	119.7 (7)	C5—C6—C7	119.7 (8)
C1—N1—Pt1	118.7 (5)	C5—C6—H6	120.1
C9—N1—Pt1	121.6 (5)	C7—C6—H6	120.1
N1—C1—C2	124.0 (7)	C8—C7—C6	121.4 (8)
N1—C1—H1	118.0	C8—C7—H7	119.3
C2—C1—H1	118.0	C6—C7—H7	119.3
C3—C2—C1	118.3 (8)	C7—C8—C9	119.5 (7)
C3—C2—H2	120.8	C7—C8—H8	120.2
C1—C2—H2	120.8	C9—C8—H8	120.2
C2—C3—C4	119.4 (8)	N1—C9—C8	120.6 (7)
C2—C3—H3	120.3	N1—C9—C4	119.0 (7)
C4—C3—H3	120.3	C8—C9—C4	120.5 (7)
			
Cl1^i^—Pt1—N1—C1	−86.2 (6)	C5—C6—C7—C8	0.3 (13)
Cl1—Pt1—N1—C1	93.8 (6)	C6—C7—C8—C9	−0.2 (12)
Cl1^i^—Pt1—N1—C9	96.8 (5)	C1—N1—C9—C8	179.3 (7)
Cl1—Pt1—N1—C9	−83.2 (5)	Pt1—N1—C9—C8	−3.7 (9)
C9—N1—C1—C2	−0.6 (12)	C1—N1—C9—C4	0.1 (10)
Pt1—N1—C1—C2	−177.7 (6)	Pt1—N1—C9—C4	177.1 (5)
N1—C1—C2—C3	0.9 (13)	C7—C8—C9—N1	−179.3 (7)
C1—C2—C3—C4	−0.6 (12)	C7—C8—C9—C4	−0.1 (12)
C2—C3—C4—C9	0.2 (12)	C3—C4—C9—N1	0.1 (11)
C2—C3—C4—C5	−179.1 (7)	C5—C4—C9—N1	179.4 (7)
C3—C4—C5—C6	179.2 (8)	C3—C4—C9—C8	−179.1 (7)
C9—C4—C5—C6	−0.1 (11)	C5—C4—C9—C8	0.2 (11)
C4—C5—C6—C7	−0.2 (12)		

Symmetry code: (i) −*x*, −*y*+1, −*z*.
